# Growth-Related Responses to the Threat of COVID-19 among Adolescents

**DOI:** 10.3390/ijerph191912597

**Published:** 2022-10-02

**Authors:** Alicja Senejko, Grzegorz Godawa, Ewa Gurba, Alicja Kalus, Krzysztof Gurba

**Affiliations:** 1 Faculty of Psychology, University of Lower Silesia, 53-611 Wrocław, Poland; 2 Department of Social Sciences, The Pontifical University of John Paul II, 31-002 Kraków, Poland; 3 Department of Philosophy, The Pontifical University of John Paul II, 31-002 Kraków, Poland; 4 Institute of Psychology, University of Opole, 45-040 Opole, Poland; 5 Institute of Journalism and International Relations, Pedagogical University of Krakow, 30-084 Kraków, Poland

**Keywords:** COVID-19 epidemic, adolescents, stress, threats, coping strategies

## Abstract

The main objective of our study was to determine whether the experience of the types of pandemic threats included in the study, could activate development responses among adolescents and what the role of the level of stress experienced during the COVID-19 pandemic is in the process of post-traumatic PTG growth. We also made an attempt to investigate whether personality traits and gender were predictors of PTG growth in adolescents. Therefore, the theoretical bases for the interpretation of the obtained results are models of post-traumatic growth (PTG), mainly by Calhoun and Tedeschi. The research was conducted in March 2020. The study subjects were 405 adolescents aged 14 to 20 years, with an average age of 17 years, of whom 59% were females and 41% were males. The following methods were used in the study: IPIP-BFM-20 to estimate five personality traits, PSS- to diagnose perceived stress, and an in-house questionnaire of pandemic threats experienced by adolescents (KŻP). The results showed that the various types of ‘pandemic’ threats (threats to life, family, and lifestyle) are positive predictors of growth-related changes; additionally, such factors as personality traits (here: extroversion), stress level, and gender had a positive mediating effect on growth-related changes. It was also possible to show that out of the four dimensions of post-traumatic growth, three could be activated under pandemic conditions. These were: changes in relationships with others, greater appreciation of everyday life, and spiritual changes. Changes in self-perception—one of the dimensions of post-traumatic growth, were not activated due to experiencing three types of pandemic threats.

## 1. Introduction

The COVID-19 pandemic is considered to be an acute and uncontrollable stressor [1] with a global impact. It poses a serious threat to the health and lives of individuals, as evidenced by statistics published by the World Health Organization. They show e.g., that more than 59,000 people died from SARS-CoV-2 infection in Poland between March 2020 and April 2021. Research reports published during this period indicate some wide-ranging psychological consequences of the COVID-19 pandemic. These include not only adults with depressive symptoms, and elevated levels of anxiety and sleep problems [2,3,4], but also adolescents and children [5,6,7,8,9,10,11,12]. Available research findings suggest that both children and adolescents, similar to adults, exhibit symptoms of depression and anxiety as well as a range of stress-related reactions, including post-traumatic stress disorder PTSD [1,6,13,14]. Pandemic living conditions are particularly disadvantageous in terms of development for adolescents and young adults. Adolescents experienced deficits with regard to meeting their vital needs under pandemic conditions; additionally, a negative impact of the pandemic on their physical and mental health, and learning opportunities has also been reported [1,15,16,17,18]. The negative impact of the pandemic on the psychological development and well-being of adolescents and young people is associated with several factors. These include the dynamics of their biological development linked to the particular sensitivity of the body, especially the brain, to stressful stimuli [19]. Furthermore, the adolescent period means an extremely high demand for peer contact, engagement in physical activity, social interaction, and similar behaviours that are normally a daily routine; however, during the pandemic, these routine forms of behaviour were extremely severely restricted due to the lockdown [15,16,20,21,22,23]. For many adolescents, being forced to stay at home and communicate mainly through social media could also imply limitations to their pursuit of autonomy, i.e., one of the basic needs in this period of life [23,24] and result in conflicts within the family. Indeed, misunderstandings and even serious conflicts may arise during the lockdown in many families, especially those in which the relationship between parents and children and between siblings was not positive [25]. As demonstrated by some research results [16,20,25,26,27], the outbreak of the pandemic and related circumstances may also cause adolescents to experience chronic stress related to concern for their own and their family’s life and health, fear for the family’s economic future, its material security, or anxiety about their own future in a country in recession, etc. We believe that the factors listed above and combined with the negative impact of the pandemic on the psychological development and well-being of teenagers and young people can be defined in terms of three groups of threats, i.e., threats to life, family, and lifestyle.

The conclusion from the analysis of previous studies on the psychological consequences of stress can be formulated as follows: most researchers treat the COVID-19 pandemic as a difficult, stressful, and even traumatic situation. It results in emotional disorders, states of depression, sleep disorders, and even post-traumatic stress disorder PTSD, as well as fear, anxiety, and a sense of threat associated with the blocking or hindering of life activities that are important for teenagers and young people [20,27,28,29]. Little is known so far about whether any positive consequences of the pandemic should be expected. The few available studies suggest that teenagers and young people can function beneficially even under pandemic conditions due to such traits as resilience, a protective factor for anxiety, depression, and stress symptoms, as well as an active coping style involving positive appraisal and thinking, distancing, problem-solving and help-seeking [1,6]. These predispositions can enhance mental health by promoting an individual’s sense of control over a chaotic environment and creating opportunities for satisfying relationships with a support network. At the same time, some differences between genders can be noted, in favour of men, in coping with highly stressful conditions using e.g., resilience [1,30,31]. The rare studies also indicate that adolescents appreciate the support of peers, and parents, and the importance of intimate relationships in coping with the hardships of life during the pandemic [6,8,9,20]. 

However, there is a lack of reports showing the feasibility of taking advantage of the pandemic conditions for adolescents’ psychosocial development. The present study was intended to fill this gap. 

We surmised, and tested this in our study, that the pandemic situation may result in psychological development along the lines of post-traumatic growth. More specifically, we were interested in whether the types of experienced pandemic threats that we took into account in our study (Threats to Life, Family, and Lifestyle) could trigger growth-related responses in the adolescents in the study. It was important for us to learn about the connections between the type of threats experienced, the strength of the stress and the personality traits (here: extroversion) on the one hand, and the adolescents’ growth-related responses which are, e.g., characterised by Tedeschi and Calhoun [32,33] in their model of post-traumatic growth (PTG) on the other hand.

### 1.1. Post-Traumatic Growth (PTG) and the Pandemic Situation

As in the PTG model, we hypothesise that growth changes may include the areas of interpersonal relationships, self-perception, and life philosophy [32,34,35]. It was this multidimensional representation of PTG, assessable in the behaviour of the adolescents in the study who experienced pandemic threats that interested us. In the domains of Personal Strength and New Possibilities, PTG refers to a sense of greater independence and resourcefulness and to discovering and pursuing new paths in life. Then, in the domain of Relating to Others, PTG includes developing greater compassion and/or closeness with other people. Another aspect, i.e., Appreciation of Life, involves perceiving the value of everyday life. The last domain of PTG, i.e., Spiritual and Existential Change, concerns the sense of a better, deeper understanding of spiritual matters and a greater sense of harmony with the world [34,35].

### 1.2. Personality Factors: Extroversion and the Epidemic Context

The results of the research conducted so far on the links between personality traits and functioning in terms of growth after severe stress are not stable or conclusive. Research reports show different sets of traits associated with PTG [36,37,38,39].

Due to the aforementioned social and normative aspects of adolescents’ experience of enhanced hygiene regimes due to COVID-19, especially the significant reduction in social contact, we focused on extroversion in our study. Extroversion represents the tendency to be sociable, active, and dominant, and heightens positive reactions from others [40,41]. On the one hand, it is associated with creativity and activity [42]. Thus, it can be predicted that extroverted individuals will make varied use of the new methods of remote communication and maintaining relationships. On the other hand, the pandemic situation may motivate extroverted adolescents to rethink the role of relationships with others and to explore new aspects and forms of social life which are important aspects of PTG [25]. For these reasons, we expect that extroversion may be a predictor of PTG growth in adolescents and a mediator between the type of pandemic threats experienced by adolescents and PTG.

### 1.3. The Role of Stress in the Growth Process

The level of stress and the fear of contagion are some of the main psychological characteristics of any pandemic, as well as an emotional component of traumatic situations [32,33]. A meta-analysis by Helgeson et al. [43] suggests that significant changes in the perception of the self and the world, shattered by the trauma being experienced may be positively related to the level of stress in the initial stages of coping with a severe threat. Butcher et al. [44] point to the adaptive value of moderate levels of stress as it helps in preparing for a possible threat and can improve learning and performance. Anxiety and excessive stress caused by a lack of data awareness are problematic as the state of non-directed agitation does not generate a rational response [45]. In the case of pandemic fear, however, the threatening stimulus is not very specific (e.g., due to the variety of possible personal and social consequences) and is located both in the present and in the future. Therefore, due to the described specificity of the level of stress and fear of SARS-CoV-2, it is difficult to predict whether the level of stress experienced by the adolescents in the study will be moderate and whether it will favour PTG.

### 1.4. Present Study

Our study was conducted in the spring of 2020, when strict epidemic restrictions were in place, in particular regarding the minimization of social contacts. Schools and universities were closed and teaching and studying took place online. Various public institutions were closed. It was not possible to meet in larger groups, neither at home nor in public places. The number of people who could stay in the store at the same time was limited, restaurants, pubs and cafes—places where young people previously visited—were closed. You had to wear a mask in public places. Our research was aimed at examining how these limitations influenced the psychosocial functioning of adolescents.

We sought answers to several research questions. 

The first question was whether the types of pandemic threats taken into account in the study could, paradoxically, activate growth responses among the adolescents in the study. 

The second research question was: which individual predispositions increase the chance of such growth? Taking into account personality predispositions, we predicted (H1) that extroversion is a predictor of PTG growth in adolescents. As hinted at above, this is due to greater motivation and ability to trigger and put more effort into new forms of contact by those with the trait of sociability. The third research question concerned the role of the level of stress in the process of post-traumatic growth experienced during the period of enhanced hygiene regime. In our study we additionally examined (question four), the role of gender in triggering growth responses in the pandemic situation.

In the case of the first, third, and fourth research questions, we did not specify the predictions in the form of hypotheses due to the lack of sufficient knowledge in this regard; our research was mainly of an exploratory nature.

## 2. Methods

### 2.1. Participants

Participants were 405 young people aged between 14 and 20 years, with an average age of 17 years, of whom 59% were women and 41% were men. Participants were pupils of four secondary schools from Krakow, 59% of them live in a big city and 41% in a small town or the countryside.

Of all the respondents, 81% live in couple families, 11% live with their mothers, and the remaining 8% live either with their fathers, grandparents, or siblings or alternately with their mothers and fathers.

### 2.2. Procedure

The survey was conducted in April 2020, during the lockdown, after schools were closed and lessons were delivered remotely, which affected the survey procedure. The questionnaire sheet for completion was posted on the websites of five Krakow secondary schools with the permission of the head teachers of those schools. It was available to students for three weeks. Parents consented to the survey of young people. The survey was voluntary and anonymous. The research ethics committee of the university, which was the main organiser of the study, agreed to conduct the study.

The study used four questionnaires and demographics to collect basic demographic data details of the subjects. The following methods were used:

### 2.3. Post-Traumatic Growth

The Post-traumatic Growth Inventory (PTGI) was used to measure post-traumatic growth [32] in Polish adaptation by Ogińska-Bulik and Juczyński [46]. The PTGI contains 21 items and is intended to measure changes in four areas of PTG (in the Polish adaptation of the method): Changes in Self-Perception (CSP), Relating to Others (RTO), Appreciation of Life (AOL), and spirituality (Spiritual and Existential Change, SEC). The respondents rate the statements given in the PTGI on a 6-point scale from 0 (no change as a result of the traumatic experience) to 5 (change to a very large extent). Cronbach’s alpha for the Polish adaptation of the PTGI ranged in the individual scales from 0.63 to 0.87.

### 2.4. Personality

The Short IPIP-BFM-20 questionnaire was used for measuring the Big Five [47], is the shortened Polish version of the IPIP-BFM-50 developed by Goldberg to estimate personality traits. The questionnaire consists of 20 items making up 5 scales (extroversion, agreeableness, conscientiousness, emotional stability, and intellect). Each of the items contains a description of the behaviour and was evaluated by the respondents on a scale from 1—strongly disagree to 5—strongly agree. The Extraversion Scale (EXT), which was taken into account in our study, measures the level of activity, energy as well as sociability, and social self-confidence (assertiveness). Reliability of the scales measured by Cronbach’s alpha in the group of Polish respondents aged 16 to 80 years was satisfactory for each scale and was in the range (0.76–0.86).

### 2.5. Stress

The Perceived Stress Scale (PSS) 10, by Cohen et al. [48], in Polish adaptation by Juczyński and Ogińska-Bulik [49] was used as a measure of global stress (LOS—Level of Stress) in a particular life situation and of coping difficulties and intensity of negative emotions over the past month. It consists of 10 statements. The answers are rated on a 5-point Likert scale (from 1—never to 5—very often). The reliability of the scale in our study was Cronbach’s alpha = 0.89.

### 2.6. COVID-19-Related Threats

Questionnaire on Types of COVID-19-related Threats, Part I (KRZC-I), by Godawa et al. [50] consists of 18 items describing pandemic-related situations that might be perceived as potentially threatening by adolescents. The subject rated the items on a 5-point scale to indicate the extent to which each of the described situations related to the COVID-19 pandemic was troublesome and threatening to them (from 1—not at all threatening to 5—threatening to a very large degree). Using an exploratory principal components factor analysis (PCA) conducted on 15 items; three factors (five items per factor) were identified as types of pandemic-related threats: 1. Life Threats (LT), which include the sense of threat to the health and life of loved ones; loss or threat of loss of job by the parents, etc. 2. Family Threats (FT), which concern frustrations due to overcrowded homes, conflicts with parents, siblings, etc. 3. Lifestyle Threats (LST), which concern the lack of opportunities to meet important life needs related to direct contact with peers, free movement and the implementation of routine, life-stabilizing behaviours. The reliability of the KRZC-I was satisfactory (FT Cronbach’s alpha = 0.79; LT Cronbach’s alpha = 0.80; LST Cronbach’s alpha = 0.78). χ^2^(19) = 187.07, *p* < 0.001, SRMR = 0.04, RMSEA = 0.086, CFI = 0.94. Factor loadings: in the range between 0.32 and 0.78.

## 3. Results

To answer the research questions and to verify hypothesis H1, an analysis was conducted using the Structural Equation Modelling (SEM) method. The analysis concerned the effect of explanatory variables, i.e., the three types of threats (FT, LT, and LST), on the explained variables: changes in relating to others (RTO), greater appreciation of life (AOE), and spiritual changes (SEC). The mediating variables were stress level (STR), gender (GEN), and extroversion (EXT). In the first step, descriptive statistics for the variables (Table 1) and correlation coefficients (Table 2) were calculated using IBM SPSS Statistics. Given that all variables showed slight deviations from the normal distribution (Kolmogorov-Smirnov test), with the critical statistic of the multivariate normal distribution not exceeding 2, an algorithm based on the parametric method of maximum likelihood (ML) was used to calculate and estimate the coefficients in SPSS AMOS 26.0. As can be seen from the analyses, the variables taken into account were characterised by satisfactory reliability measured by Cronbach’s Alpha method. It is also worth noting that out of the three types of pandemic threats, the young people in the survey experienced lifestyle threats (LST) most intensely, and life threats (LT)—slightly less intensely. As far as the post-traumatic growth (PTG) variables are concerned, the most frequently mentioned were changes in relating to others (RTO) and AOL—a greater appreciation of life. Less frequently respondents indicated spiritual changes (SEC) while, interestingly, changes in self-perception (CSP) did not prove to be significantly represented in the survey presented. 

The correlation analysis (Table 2) shows that all three types of threats had positive, weak, or moderate, significant correlations with each other. EXT extraversion was correlated positively and weakly or moderately with the two threats, i.e., LT and LST. Further correlations were also noteworthy. As it turned out, LOS was correlated positively only with threats: most strongly with SLT, somewhat less strongly with FT, and least strongly but significantly with LT. Regarding factors related to PTG, strong positive correlations should be noted between all the three variables relevant to our study, i.e., RTO, AOL, and SEC. Furthermore, greater appreciation of relationships with others (RTO) was significantly and positively, with weak or moderate strength, correlated with LST, LT, EXT, and LOS. On the other hand, a greater appreciation of everyday life (AOL) was significantly but rather weakly correlated with all the three types of threats (most strongly with LT) as well as with EXT and LOS. The last of the analysed variables, spiritual changes (ESC), was weakly and positively correlated with LT and EXT (Table 2).

### Path Analysis Results

**Figure 1:**
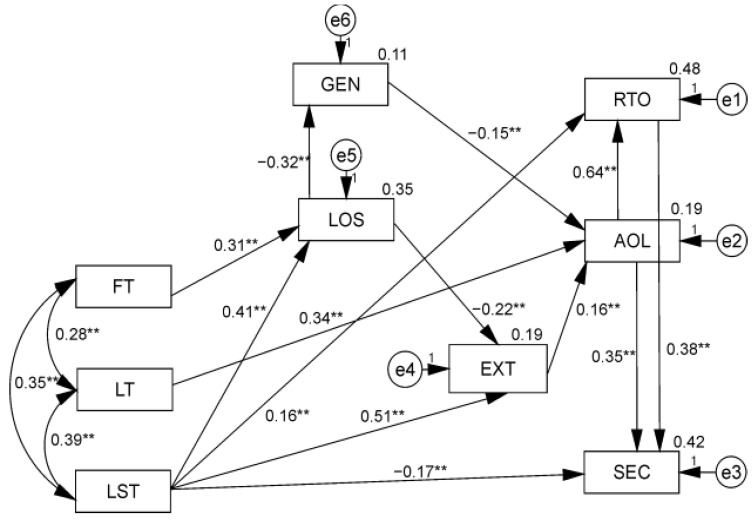
Results from structural equation modelling for the effects of pandemic threats (FT, LT, and LST) on dimensions of post-traumatic growth (changes in RTO, AOL, and SEC), with mediating effects of EXT, LOS, and GEN (standardised coefficients). *Note*: All parameters are significant at the *p* < 0.01 level **.

A diagram showing relations between the variables along with parameter values is shown in Figure 1. The model is a very good fit to the data (χ^2^/df = 1.43, RMSEA = 0.033 (0.000–0.058), GFI = 0.984, AGFI = 0.965, TLI = 0.984, CFI = 0.99). The model explains 42% of the variance in SEC, 48% of the variance in RTO, 35% of the variance in LOS, and 19% of the variance in AOL. Regarding the three explanatory variables, i.e., the pandemic threats, as theoretically hypothesised, LST, LT, and FT had positive and significant correlations with each other but low enough to be considered separable.

In particular, LST had a multifaceted effect, direct and indirect, on the explained variables. Thus, LST directly contributed both to positive changes in RTO (a weak positive predictor) and to attenuating SEC (a weak negative predictor). Interestingly, LST indirectly enhanced SEC through changes in RTO. LST also contributed indirectly, through EXT, GEN, and LOS, to strengthening another explained variable related to post-traumatic growth, i.e., AOL. Thus, they were positively correlated with EXT, which in turn was a weak positive predictor of AOL. Additionally, LST enhanced AOL, especially in women, by enhancing LOS. A corresponding pattern of mediating effects on AOL (through GEN and LOS), was found for another explanatory variable, i.e., FT.

The third explanatory variable, i.e., LT, had a direct positive effect on AOL (a positive and moderate predictor), and through this explained variable it had a strong effect on both RTO and (somewhat weaker, positive) on SEC, the other explained variables. 

Gender had a weak indirect effect on the severity of relationship changes in RTO and favoured SEC through enhanced AOL (higher scores were associated with women). 

EXT had a weak positive effect on AOL and, through this variable, on the other explained variables, i.e., the level of change in RTO and SEC. 

As we have shown above, LOS was an important mediating factor between the impact of two of the three pandemic threats (FT and LST, both of which increased stress levels) on the explained variables, i.e., the types of post-traumatic growth. Importantly, LOS did not have a direct impact on PTG but through further mediating variables. Firstly, through GEN (female respondents had higher levels of stress and it was they who were more able than men to appreciate life more (AOL) and to develop other dimensions of PTG in the pandemic situation). Secondly, LOS had an indirect influence on PTG through EXT (lower levels of stress increased the positive effect of EXT on AOL and, through this variable, on the other relevant dimensions of post-traumatic growth of RTO and SEC).

As it turned out, the correlation between the explained variables themselves also had an interesting pattern. A higher level of SEC was both the result of a direct effect of AOL, i.e., it increased as the subjects’ appreciation of life increased, and it was also directly impacted by the positive changes in RTO, which in turn was additionally positively impacted by AOL. Thus, increased AOL directly as well as indirectly (through a positive effect on RTO) influenced SEC (Figure 1). 

## 4. Discussion

The main goal of our study was to determine whether the experience of the types of pandemic threats included in the study can activate post-traumatic growth among the studied adolescents and what is the role of the level of stress, personality (here: extraversion) and gender in this process. COVID-19-Related Threats, this designation identifies categories of conditions related to the pandemic that the adolescents surveyed experienced as difficult and threatening to them, their families, and also to their lifestyle.

The attention of clinical and health psychologists, and more recently also of developmental psychologists, has been increasingly focused on the positive changes that can be experienced by a person struggling with a potential traumatic life event [51,52,53,54,55]. A pandemic, i.e., health- and even life-threatening situations for ourselves and loved ones, can be seen as at least a potentially traumatic life event. The threats that we considered in the study were threats to family (FT), life (LT), and lifestyle (LST). The explained variable comprised four dimensions of post-traumatic growth (PTG), three of which appeared to play a significant role in this process. These were spiritual and existential change (SEC), greater appreciation of life (AOL), and positive changes in relating to others (RTO). Non-significant correlations between the variables estimated in the study were obtained for the dimension of post-traumatic growth referred to as Changes in Self-Perception (CSP), which, as we remember, in the Polish adaptation of the method measuring PTG comprised two parameters from the basic version of the method, i.e., Personal Strength, and New Possibilities [35,56]. It should be specified that due to the variability of the pandemic situation, we treated PTG as an initial, dynamic process rather than a relatively permanent outcome. As can be surmised, the initial stage of the pandemic during which the Spring 2020 study was conducted, and the sense of threat associated with it, provoked reflection and functioning focused more on issues concerning interpersonal relationships, the role of other people in our lives (i.e., RTO) and appreciation of life itself in its every day, ‘ordinary’ aspects (AOL), or reflection on life, fate, chance, the role of values, religion and/or faith in our lives (i.e., SEC). Our research has shown that the pandemic situation, especially the lockdown, i.e., forced restriction of activity and movement, was not conducive to a sense of greater independence and resourcefulness or to discovering and pursuing new paths in life by the teenagers and adolescents in the study, which characterised the fourth dimension of post-traumatic growth, i.e., Changes in Self-Perception, CSP [32]. This is particularly true with regard to the age group in question that such growth changes require some free space in the physical and symbolic sense, in which young people can express their autonomy and undertake a variety of exploratory activities allowing them to test themselves in different life circumstances and thus gain greater insight into themselves, their own preferences, needs, etc. [24,57,58]. It is usually in this way that adolescents shape their identity while preparing to make important commitments that will give direction to their lives, to pursue in the future the goals and values that are important to them. The situation at the beginning of the pandemic not only did not facilitate such explorations but actually prevented them [59].

The results of our research justified a positive answer to the first research question, which concerned whether the types of pandemic threats taken into account can activate growth-like reactions of the adolescents in the survey. As it turned out, all three types of threats proved to be significant predictors of the three—out of four—dimensions of post-traumatic growth mentioned above. In particular the LST, i.e., blocking or strongly impeding the possibility of pursuing behaviours through which young people used to satisfy their needs, passions, and life values, had in this respect an interesting impact on the explained variables. LST directly contributed both to positive changes in RTO and also indirectly, through EXT, LOS, and GEN, to a greater AOL. But experiencing LST did not directly contribute to increasing SEC but conversely, directly contributed to weakening it; only indirectly did LST strengthen SEC through changes in RTO and greater AOL. Moreover, also the other two types of pandemic threats, LT and FT, had no direct positive effect on spiritual changes. LT, which was associated with the disturbed physical and material security of the respondents’ and their loved ones’ existence, had a direct positive effect on AOL and only through this factor—on the other two significant PTG variables, i.e., RTO and SEC. In contrast, FT, associated with possible conflicts in the family and lack of understanding on the part of family members, only indirectly (through LOS and GEN) interacted with AOL and only through this factor—with the other variables, RTO and SEC. Thus, it can be concluded from the results of our study that the typical path of spiritual change for adolescents is through relationships with others and concrete actions in the world; and it is only through these that significant spiritual changes are brought about [59,60]. It can also be assumed that a longer period of time than was the case in our study is necessary in order to initiate in the adolescents in question an autonomously running process of spiritual changes referred to in the model of PTG, without the mediation of the other two dimensions of PTG (changes in relationships and greater appreciation of life). For adolescents, spiritual changes are not easy and often take place within religious identity crises, with the presence of ambivalent religious attitudes [61,62]. They are also important in adolescents’ coping with stressful situations [63]. Indeed, spiritual change is mainly a cognitive process in which those who experienced trauma applied positive interpretations and found meaning in traumatic events, reflected in a sense of a better, deeper understanding of spiritual matters, the sacred, and a greater sense of harmony with the world [35,64,65]. 

The second research question concerned individual predispositions that could increase the chance of post-traumatic growth. Considering personality predispositions, we predicted (H1.) that EXT is a predictor of PTG in adolescents. Admittedly, some believe [66] that with regard to such highly stressful situations as the COVID-19 pandemic less space should be given to dispositional tendencies as compared with situational characteristics in predicting human behaviour. There are studies addressing the links between personality traits and growth-related forms of PTG functioning after severe, even traumatic stress [40,51]. While these are not stable results, it was possible to distinguish from them certain Big Five personality traits that appear more consistently than others in various studies of potential predictors of PTG as positively correlated with post-traumatic growth. These traits included extroversion [32,36,37,67,68]. Our research confirmed these results. The hypothesis H1. that we formulated was positively verified. As it turned out, EXT was a direct predictor of AOL and, through this factor, influenced the other PTG variables, RTO and SEC. In addition, EXT mediated the effects of two of the three epidemic threats, i.e., LST and FT, on dimensions of post-traumatic growth. Both these types of threats are closely related to entry into social relations. We highlighted the trait of extroversion because of the social aspects of the enhanced hygiene regime due to COVID-19. One of the main restrictions was, as you know, limiting social contacts. While complying with this requirement was tough for everyone, it may have been a particularly big challenge for extrovert young people [66,67,68,69]. However, as we rightly predicted, by redefining social interactions as health- and life-threatening the pandemic situation may also motivate individuals, especially extroverts, to reflect on the role of relations with others, compassion, and appreciation of the value of everyday life, which are important aspects of post-traumatic growth [59,66].

Our third research question concerned the role of the LOS in the process of post-traumatic growth experienced during the pandemic period. As it turned out in the light of our research, LOS was an important mediating factor in the impact of variables on the dimensions of post-traumatic growth. First, it mediated between the effects of pandemic threats, FT and LST, which increased LOS, on the explained variables, i.e., types of post-traumatic growth. Importantly, LOS did not interact directly with PTG dimensions but through the mediating variables. Firstly, through GEN (female respondents had higher levels of stress and it was they who were more able than men to appreciate life more (AOL) and to develop other dimensions of PTG). Secondly, LOS indirectly influenced PTG through EXT (lower stress level increased the positive effect of EXT on AOL and, through this variable, on the other relevant dimensions of post-traumatic growth). 

The results concerning the role of LOS as a mediating factor between the type of pandemic threats and PTG-related changes in the functioning of the adolescents in the survey may indicate that the level of stress experienced by adolescents is not among the highest ones that prevent the course of the growth processes but, on the contrary, contributes to their activation, and therefore probably has at most a medium level of intensity. If this is the case, it may constitute an additional factor triggering and influencing the quality of cognitive processing involved in the process of post-traumatic growth changes [43,44,70]. Our research has therefore shown that the level of stress experienced by adolescents under pandemic conditions may be a component of the process that motivates an individual to engage in reflections leading to PTG and mediates the relation between the type of pandemic threats experienced by adolescents and the dimensions of post-traumatic growth. 

In our study, we additionally examined (question four) the role of gender in activating growth responses in the pandemic situation. The results of previous research on the association between gender and the experience of growth changes after a powerful and traumatic life event are particularly inconclusive, especially with regard to children and adolescents [1,39,55,65]. However, our research clearly showed that it was women, more specifically female teenagers and adolescents, rather than men or boys who took advantage of the pandemic situation to make growth-related changes in their own functioning. This was especially true in terms of a greater appreciation of everyday life (AOL) and through this factor, increased changes in RTO and SEC. This outcome might be due to the specifics of parenting and the social expectations towards daughters in Poland, especially on the part of their parents. They expect their daughters to be educated women with challenging goals in life. In Poland, this is manifested, e.g., in the fact that the number of women with higher education has exceeded the number of men with the same higher education in Poland for many years now [71]. Such social expectations may, perhaps, translate also into the relatively good coping of young Polish women in life and their development potential, including post-traumatic growth.

## 5. Conclusions 

The research into adolescents’ growth responses to the threats of the COVID-19 pandemic proved, in our view, to be a very relevant research task. The results showed that the pandemic threats (to life, family, and lifestyle) were positive predictors of growth-related changes; at the same time, factors such as personality traits (here: extroversion), level of stress, and gender also had mediating positive effects on growth-related changes. It was also possible to show that three of the four dimensions of post-traumatic growth could be activated under pandemic conditions. These were Relating to Others, Appreciation of Life, and Spiritual and Existential Change. One dimension of post-traumatic growth, on the other hand, was not activated under conditions of experiencing the three types of pandemic threats. This was the dimension of Changes in Self-Perception, manifested in the sense of greater independence and resourcefulness, personal strength, and greater self-confidence. It appears that the young people in the study did not have the opportunity to activate/shape these characteristics in themselves, due to both the specificity of the pandemic circumstances themselves (lockdown, limited mobility, etc.) and the developmental specificity of the subjects’ period of the life, i.e., adolescence. We hope that through our research it will be possible to gain a deeper, more complete understanding of the phenomenon of post-traumatic growth occurring in young people also under pandemic conditions. This may open new research areas for researchers and provide educators and therapists with new ideas for strategies for working with young people who experience highly threatening, traumatic life events. 

Our research has shown that for young people a very important factor, both in terms of experiencing strong threats and reacting to them in the form of post-traumatic growth, are social relationships, mainly peer-to-peer, involving direct contact and related to the style of communicating with friends, on the shape of rituals of meetings and contacts, and not—as is commonly believed—in a form mediated by social media. Blocking such contacts may be experienced by young people as a strong threat, even a trauma. For parents, educators and therapists, the most important conclusion from our research may be important to take into account in their contact with a young person, which can be reduced to the statement that the adolescent develops mainly through social contacts in their ritualized, direct dimension; they support his sense of value and deepen his sense of identity, provide a sense of security, and are also the basis for the processes that make up post-traumatic growth.

Our research, as with any research, also has its limitations. These include, first of all, the fact that self-report research methods were used online, which means that some responses, especially from adolescents, might have been of a self-presentational nature. Another specific limitation was the characteristics of the study sample. On the one hand, we conducted the research with the participation of several hundred adolescents, but on the other hand, they were all from the same location, a large city in Poland. The respondents were in different stages of growing up (aged 14 to 20); however, due to the small sample size, we have not decided to carry out analyses taking into account the development stage. Therefore, we are far from recommending that the results obtained by us relate to the entire population of young people in Poland, or treat them as representative of the adolescence period. For the above reasons, as well as because of the relatively small group of respondents we are rather far from recommending that the results we obtained to be applied to the entire population of adolescents in Poland or be perceived as representative of the period of adolescence.

## Figures and Tables

**Table 1:** Descriptive statistics of variables (N = 405).

	*M*	*SD*	Sk	Kurt	*S*-*K*	α
FT—Family Threats	2.20	1.00	0.59	−0.49	0.12 **	0.79
LT—Life Threats	2.96	0.99	0.13	−0.69	0.07 **	0.80
LST—Lifestyle Threats	3.27	0.96	−0.38	−0.59	0.10 **	0.78
EXT—Extraversion	3.07	1.05	−0.14	−0.86	0.09 **	0.84
RTO—Relating to Others	2.28	0.94	0.52	−0.42	0.09 **	0.85
AOL—Appreciation of Life	2.28	1.01	0.55	−0.50	0.12 **	0.73
SEC—Spiritual and Existential Change	1.87	1.05	1.06	0.26	0.26 **	0.77
LOS—Level of Stress	3.03	0.80	−0.11	−0.49	0.05 *	0.89

* *S*-*K* < 0.05, ** *S*-*K* < 0.01

**Table 2:** Correlation matrix (Pearson’s r) for research indicators (N = 405).

		1	2	3	4	5	6	7
1	FT							
2	LT	0.28 **						
3	LST	0.35 **	0.39 **					
4	EXT	0.06	0.10 *	0.39 **				
5	LOS	0.45 **	0.28 **	0.52 **	0.04			
6	RTO	0.01	0.25 **	0.34 **	0.26 **	0.12 *		
7	AOL	0.11 *	0.38 **	0.29 **	0.21 **	0.11 *	0.68 **	
8	SEC	−0.01	0.19 **	0.06	0.12 *	−0.02	0.57 **	0.57 **

FT—Family Threats, LT—Life Threats, LST—Life Style Threats, EXT—Extraversion, LOS—Level of Stress, RTO—Relating to Others, AOL—Appreciation of Life, SEC—Spiritual and Existential Change. * *p* < 0.05, ** *p* < 0.01.

## Data Availability

Not applicable.
